# AGE DIFFERENCES IN SOCIAL NETWORK PREFERENCES: IMPLICATIONS FOR DECISION MAKING

**DOI:** 10.1093/geroni/igad104.0098

**Published:** 2023-12-21

**Authors:** Tess Wild, Corinna Löckenhoff

**Affiliations:** Cornell University, Ithaca, New York, United States; Cornell University, Ithaca, New York, United States

## Abstract

Many consequential decisions are made in the context of a social network, but it is not clear how lifespan developmental changes may influence the way people integrate others into their decision-making processes. Research shows that, compared to younger adults, older adults perform less exhaustive pre-decisional information searches, have smaller and more close-knit social networks, and place more value on harmonious relationships (e.g., Hess et al., 2015). Drawing on these findings, a pre-registered survey study examined age differences in the size, composition, and utilization of decision-related social networks as well as subjective goals and perceptions regarding joint decisions. An adult lifespan sample (N = 432; M age = 50.9375; SD age = 19.53267, range age = 18-96 years) considered hypothetical decisions involving specific (apartment, car, physician, and insurance plan) and generic (important vs. minor) scenarios. As predicted, linear regressions revealed that compared to younger respondents, older respondents reported that they would consult fewer people fewer times, and they were less likely to recruit distant social partners or professionals (ps<.05). Contrary to predictions, the tendency to prioritize relationship harmony over decision quality was negatively associated with age (p<.05). Further, there was no significant relationship between age and the perceived helpfulness of involving others. Findings remained robust when controlling for scenario type and demographic characteristics (gender, education level, race, income). This indicates that the size, composition, and utilization of decision-related social networks differs by age. Further research is needed to examine potential implications for decision quality and satisfaction.

